# Effects of Insect Protein Supplementation during Resistance Training on Changes in Muscle Mass and Strength in Young Men

**DOI:** 10.3390/nu10030335

**Published:** 2018-03-10

**Authors:** Mathias T. Vangsoe, Malte S. Joergensen, Lars-Henrik L. Heckmann, Mette Hansen

**Affiliations:** 1 Section for Sport Science, Department for Public Health, Aarhus University, Dalgas Avenue 4, 8000 Aarhus, Denmark; major.malte@gmail.com (M.S.J.); mhan@ph.au.dk (M.H.); 2 Danish Technological Institute, Life Science Division, Kongsvang Allé 29, 8000 Aarhus, Denmark; lhlh@teknologisk.dk

**Keywords:** insect, hypertrophy, nutrition, resistance training, buffalo larvae

## Abstract

During prolonged resistance training, protein supplementation is known to promote morphological changes; however, no previous training studies have tested the effect of insect protein isolate in a human trial. The aim of this study was to investigate the potential effect of insect protein as a dietary supplement to increase muscle hypertrophy and strength gains during prolonged resistance training in young men. Eighteen healthy young men performed resistance training four day/week for eight weeks. Subjects were block randomized into two groups consuming either an insect protein isolate or isocaloric carbohydrate supplementation within 1 h after training and pre-sleep on training days. Strength and body composition were measured before and after intervention to detect adaptions to the resistance training. Three-day weighed dietary records were completed before and during intervention. Fat- and bone- free mass (FBFM) improved significantly in both groups (Mean (95% confidence interval (CI))), control group (Con): (2.5 kg (1.5, 3.5) *p <* 0.01), protein group (Pro): (2.7 kg (1.6, 3.8) *p <* 0.01) from pre- to post- leg and bench press one repetition maximum (1 RM) improved by Con: (42.0 kg (32.0, 52.0) *p <* 0.01) and (13.8 kg (10.3, 17.2) *p <* 0.01), Pro: (36.6 kg (27.3, 45.8) *p <* 0.01) and (8.1 kg (4.5, 11.8) *p <* 0.01), respectively. No significant differences in body composition and muscle strength improvements were found between groups. In young healthy men, insect protein supplementation did not improve adaptations to eight weeks of resistance training in comparison to carbohydrate supplementation. A high habitual protein intake in both Con and Pro may partly explain our observation of no superior effect of insect protein supplementation.

## 1. Introduction

The United Nation’s (UN’s) Food and Agriculture Organization (FAO) estimates that global food production must increase by >70% by 2050 to feed the growing world population, expected to reach 9–10 billion people [1]. This highlights the importance of generating new and sustainable systems for production of animal-based food that provide an excellent source of protein for human nutrition. Protein from insects has great potential for being a climate-friendly, high-quality solution to meet future protein demands. Nevertheless, the nutritional value of this protein source has not been extensively investigated [2,3]—and never from the perspective to muscle hypertrophy in humans. We therefore find it highly relevant to investigate this field regarding the potential beneficial effects of insect protein as human nutrition in a training context.

Previous investigations have shown that after resistance exercise the skeletal muscle is sensitive to protein supplementation for up to 48 h post-exercise [4,5,6,7]. Furthermore, a meta-analysis by Cermak et al. (2012) reported that protein supplementation enhances improvements in muscle mass and strength [8]. Nevertheless, a range of studies included in the meta-analysis failed to show a significant effect of protein supplementation [8]. Suboptimal timing or doses of protein may be part of the explanation. Morton et al. [9] suggest that an intake of 0.4 g/kg/meal would be the optimal dose of protein per meal to maximally stimulate muscle protein synthesis (MPS) rate during recovery from whole-body resistance exercise, and that protein ingestion beyond this dose does not elicit any further stimulation [10]. 

Areta et al. [11] propose that protein ingestion of 20 g every 3 h within the first 24 h after a single resistance exercise bout promotes the effect of MPS. In line with this, acute and training trials have shown that pre-sleep protein supplementation enhances MPS during the night and increases the adaptation to strength training in muscle mass and strength [9,12,13]. Therefore, evidence suggests that protein supplementation in a dose of 0.4 g/kg/meal ingested between main meals (after exercise and pre-sleep) may improve adaptation to resistance training in terms of muscle mass and strength.

The quality of the most commonly consumed isolated protein sources, whey, casein, and soy, differs inter alia (i.a.) with respect to digestibility and amino acid (AA) profile. A high-quality protein contains a large amount of essential amino acids (EAA) and branched-chained amino acids (BCAA), especially leucine, which, besides being a building block for proteins, also seems to have a stimulating effect on skeletal MPS [6,14]. Furthermore, the structure of proteins and thereby their digestibility seems to differ. A study by Bos et al. [15] shows that milk protein (whey and casein) conveys a more balanced pattern of amino acids to the liver compared to soy protein. Hence, supplementation with animal protein has proven to lead to greater gains in muscle mass and strength compared to vegetable sources such as soy [16]—which, however, is a more sustainable protein source from an environmental perspective. Insect protein isolate could, as a climate-friendly animal protein source, be a compromise for athletes with environmental concerns who seek to enhance muscle growth and strength. 

The aim of this study was to investigate the potential effect of insect protein isolate as a dietary supplement to increase muscle hypertrophy and strength gains during prolonged resistance training when protein dose and timing meet current recommendations. Primary and secondary outcomes are expressed as changes in fat- and bone- free mass (FBFM) and muscle strength, respectively. We hypothesize that supplementation of insect protein isolate promotes greater muscle hypertrophy and strength gains during prolonged resistance training compared to isocaloric carbohydrate supplementation.

## 2. Methods

### 2.1. Subjects

Eighteen healthy young males (age: 24.2 ± 2.6 year, body weight: 79.9 ± 9.0 kg, height: 186.6 ± 6.6 cm), with no difference between groups, were recruited via posters at Aarhus University and local dorms in Aarhus, and online via posts on Facebook. Inclusion criteria were males from the age of 18 to 30 years who had previous experience performing resistance exercise. Sport and activities up to 10 h per week were accepted, if activities did not include structured resistance training. Exclusion criteria were resistance training in the past year, the use of medication or dietary supplements, smoking and injuries, or health issues conflicting with participation in the training program. At initial contact, subjects were screened by questionnaire against criteria of inclusion and exclusion. Based on the responses to the questionnaire, subjects were deemed suitable. The trial complied with the Declaration of Helsinki and was approved by The Central Denmark Region Committees on Health Research Ethics. Clinical trial registry number: NCT03034239. All subjects gave their informed consent to participate before the experiment.

### 2.2. Protocol

The trial was designed as a randomized, controlled, single-blinded trial consisting of eight weeks of resistance training four days/week, performed as a whole-body split routine. The subjects were blinded and randomly assigned to two groups that ingested either an insect-protein bar containing 0.4 g protein/kg (Pro, *n* = 9) or an isocaloric carbohydrate bar (Con, *n* = 9) within 1 h after each training and 1 h before night sleep on training days. Before and after the intervention, strength and body composition were measured. Dietary records were obtained (three-day registration periods) before and during the intervention period.

### 2.3. Training

The subjects performed the training at Section for Sports Science, Department for Public Health, Aarhus University. Before starting the training regime, all subjects were carefully instructed on how to perform each exercise to ensure uniform execution and avoid training-related injuries. Techniques were re-evaluated in weeks three and six. Subjects were told to perform 5–10 min of light aerobic exercise before each training session [17]. 

The structure of the training program is shown in Table 1. The lower-body (LB) and upper-body (UB) sessions comprised four and seven different exercises, respectively (LB: 45° incline leg press, knee extension, leg curl, and calf raises), (UB: bench press, lying cable chest flies, lateral pulldown, seated row, biceps curl, triceps extension, and standing abdominal crunches). Subjects were required to follow the order of exercises listed above, starting with large muscle group exercises [18]. The intensity of load was 80% of one repetition maximum (1 RM) in all exercises except for the three involving only relatively small muscle groups (leg curl, lying cable chest flies, and biceps curl), where the intensity of load was 70% [19]. 1 RM loads were assessed at the first training session for both LB and UB and adjusted in weeks 3 and 6 to ensure optimal training intensity. Each training week consisted of two LB and two UB sessions. The two UB and LB training sessions had to be interspersed with at least 48 h between them [20]. From week 3 onward, the last set in each exercise was performed to voluntary failure [21]. The subjects completed a training log after each training session. The subjects were paired and performed the training sessions together to enhance compliance.

### 2.4. Dietary Supplementation

The dietary supplementations were served as baked bars. All bars contained a mix of banana, ginger, oats, raisins, and cinnamon (137 kcal; ~26.3 g carbohydrate, ~3.6 g protein, ~0.4 g fat). A dose of 0.4 g/kg/bar insect protein (Experimental Protein Isolate, Proti-Farm R&D BV, Ermelo, The Netherlands; ~82% protein) from the lesser mealworm (*Alphitobius diaperinus*) was added for the Pro group supplementation. The manufacturer stated that the total protein content of the insect protein isolate was 80–85%, evaluated on the basis of the Kjeldahl method with a nitrogen-to-protein conversion factor (Kp) of 6.25. A carbohydrate-rich mix of potato flour (348 kcal/100 g, 85 g carbohydrate) and Maxim Sports Drink ((Orkla Care, Ishoej, Denmark) (359 kcal/100 g, 90 g carbohydrate)), ratio 1:1, was added for the Con bar to match the Pro bar in terms of energy content. The Con and Pro bars were isocaloric. Thanks to the similar flavors from banana, ginger, and cinnamon, the bars tasted and smelled roughly the same.

### 2.5. Dual-Energy X-ray Absorptiometry (DXA)

Body composition was measured in the week before the intervention and two days after the last training session using whole-body Dual-energy X-ray absorptiometry (DXA) (GE Lunar DXA scan, GE Healthcare, Madison, WI, USA). The system’s software package (enCORE software v16.0, GE Healthcare, Madison, WI, USA) was used to determine changes in body composition BW (body weight), FM (fat mass), BMC (bone mineral content), and FBFM. In addition to whole-body measurements, the LB and UB regions were analyzed separately. The distinction between LB and UB was made by a line crossing the superior points of the crista iliaca. The head (line crossing chin) was not included in UB. The subjects were scanned at the same time of day at week 0 and 9 and were told to be fasting from 10:00 p.m. the night before the scan, and not to drink 2 h before. Before the scan, subjects had their weight and height determined. All scans were performed at Section for Sports Science, Department for Public Health at Aarhus University.

### 2.6. Strength Testing

The subjects underwent a series of different strength tests before and after the training period. The protocol consisted of two separate test days with 1 RM test in leg press and bench press, 80% of 1 RM to failure in leg press and bench press (day 2), isometric maximal voluntary contraction (MVC) of right leg knee extensor and flexor, and a counter-movement jump (CMJ) test (day 1). 

Before 1 RM testing, all subjects performed a familiarization test to avoid any major variation in results affected by the test situation, with a minimum of one day’s rest between the familiarization test and the valid test. Subjects were told to avoid any strenuous exercise the day prior to the test. A standardized warm-up was performed consisting of 5 min of light aerobic exercise followed by 10 repetitions at 50% of estimated 1 RM load with a 2-min rest and 3–4 repetitions at 80% of 1 RM load with a 3-min rest. The test consisted of three to five progressive 1 RM attempts interspersed with a 3-min rest [22]. When 1 RM was determined, the subjects rested for 5 min before performing a set at 80% of 1 RM to failure. The procedure was repeated for bench press. 

MVC was determined seated on a dynamometer (Humac Norm, CSMI, Stoughton, WI, USA) with 90° hip flexion and the hands were placed on the lap. All subjects performed a warm-up of 5 min light aerobic exercise (self-chosen load) on a stationary bicycle (Monark, Varberg, Sweden) before the test. Their isometric knee extensor and flexor MVC were then measured in the dynamometer by five attempts at 70° knee flexion [23] (0° equals full knee extension) and 20° knee flexion, respectively. All attempts were followed by a 90-s rest period. All trials were sampled at 100 Hz. Peak torque from the best of the trials was used for further analysis

Following the MVC, the subjects performed a CMJ test on a jumping mat (Speedmat, Swiftperformance, Wacol, Australia). Jumping height, subjects’ weight, and flight time were used to assess the maximal power output during the jump. Subjects were given five attempts with a 1-min recovery between them. The best of the five attempts were used for further analysis.

### 2.7. Dietary Analysis

Before the intervention, the participants completed a 3-day weighed dietary record on three consecutive days, including one weekend day. The data were analyzed for habitual intake of energy and macronutrients. During the intervention, a similar dietary record was performed. All dietary data (except the Con and Pro supplements) were registered in the software program (Madlog, MADLOG Aps, Kolding, Denmark). Records not meeting the criteria of Goldberg cut-off limits (energy intake/basal metabolic rate (EI/BMR) < 1.00 or >2.4) [24] were excluded from the analysis (*n* = 3).

### 2.8. Statistics

Statistical analyses were performed using the EpiBasic v3.0 (Svend Juul & Morten Frydenberg, Aarhus, Denmark) [25]. All data were checked for normality of distribution and baseline characteristics between groups were compared by means of an independent *z*-test. A two-way repeated measure analysis of variance (ANOVA) with time as a within factor and group as a between factor was used to determine any time-by-group interaction in all body composition variables, dietary variables, and strength changes. Results are presented as mean ± standard deviation (SD) with delta values shown as mean (95% confidence interval (CI)). Alpha level was set to ≤0.05.

## 3. Results

### 3.1. Subjects

**Figure 1:**
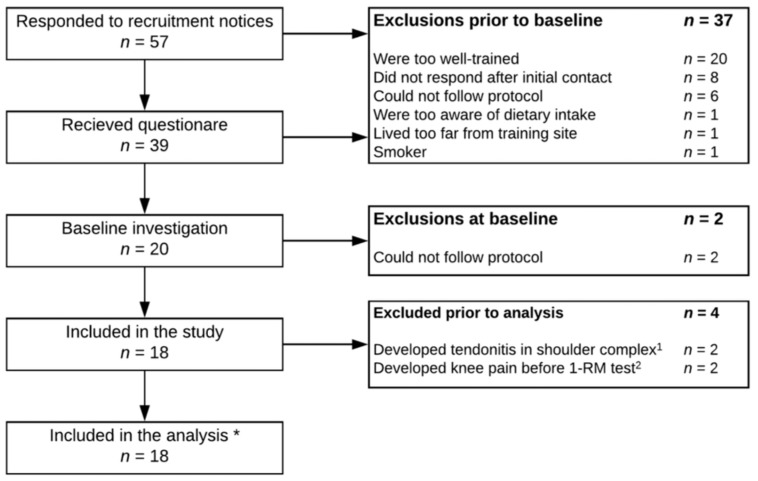
* Only 14 subjects were included in all analyses; ^1^ Lower-body data were still included in analysis; ^2^ Data from maximal voluntary contraction (MVC) test and counter-movement jump (CMJ) were still included in analysis.

All 18 subjects included in the study completed the intervention period with a training compliance of 97.6 ± 2.7%. The dietary supplementations were consumed with no major complications in both groups. Two subjects (Pro) suffered an injury in the shoulder complex from activity not related to the training protocol (Figure 1). Upper-body data from these two subjects were excluded from the analysis. During post-strength testing, two subjects (Con) suffered knee pain and could not perform the 1 RM and 80% to failure leg press.

### 3.2. Body Composition

**Figure 2:**
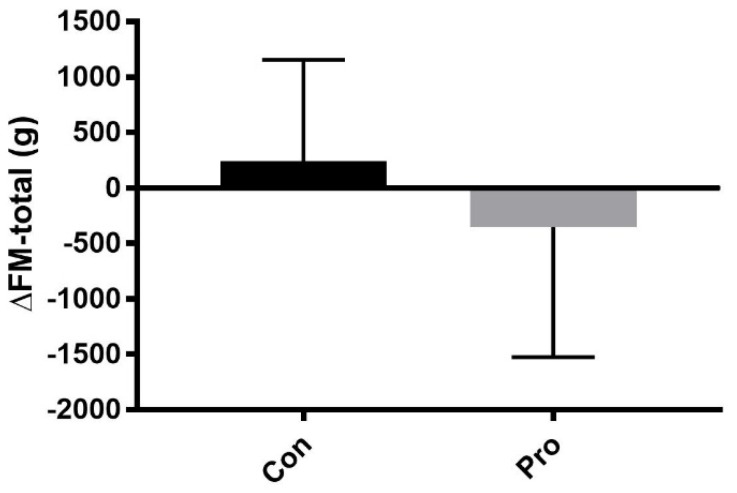
Change in total fat mass (FM) from pre-intervention (Pre) to post-intervention (Post) in control group (Con, *n* = 9) and protein group (Pro, *n* = 7). Data are shown as mean ± standard deviation (SD).

**Figure 3:**
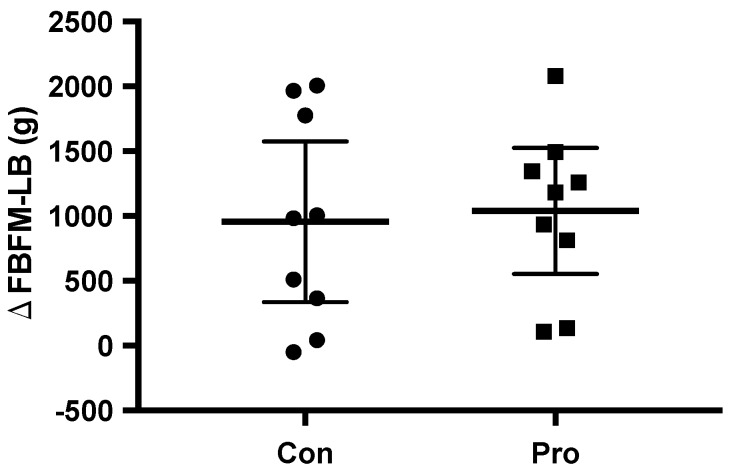
Con, control group; Pro, protein group. Individual and mean (95% confidence interval (CI)) changes in ΔLB-FBFM from pre to post. Dots represent Con (Con; *n* = 9), and boxes represent Pro (Pro; *n* = 9). .

Over eight weeks of resistance training with dietary supplementation, total body weight increased significantly in both groups (Table 2). FM increased in Con and decreased in Pro but neither showed statistical significance (Figure 2). FBFM also increased significantly in both groups, with a slight tendency towards greater gains in ΔLB-FBFM in Pro (Figure 3). The group-by-time interaction analysis showed no significant difference in any of the parameters.

### 3.3. Dietary Data

Table 3 shows the dietary intake data from Pro and Con as estimated from the subjects 3-day weighed dietary records pre- and mid- intervention. All subjects completed both registration periods, but data not meeting Goldberg’s cut-off limits (EI/BMR > 1.00 or <2.4) [24] were excluded from the analysis (*n* = 3, Con).

Con increased total energy, protein and carbohydrate intake by 30%, 26% and 43% (*p <* 0.05) from pre- to mid- intervention, while Pro increased protein intake by 48% (*p <* 0.001). No significant difference in total energy and fat intake were observed between groups. However, there was a tendency towards a higher energy intake in Con compared to Pro (*p* = 0.10) from pre- to mid-intervention. Furthermore, a significant time-by-treatment interaction for protein intake in Pro (*p <* 0.05) and carbohydrate intake in Con (*p <* 0.05) was evident.

### 3.4. Strength

Changes in 1 RM, leg- and bench- press volume, MVC knee extension (Ext.), knee flexion (Flex.) and CMJ between pre- and post- intervention are shown in Table 4. Leg and bench press volume were calculated as repetitions times load at 80% of 1 RM to failure. Both groups showed significant improvements in all strength tests from pre to post (*p <* 0.05), except bench press volume Pro (*p* = 0.54) Con (*p* = 0.97) and leg press volume Pro (*p* = 0.08). However, a tendency towards a time effect for leg press volume for Pro was observed. No difference in group-by-time interaction was detected at any point.

### 3.5. Training Volume

Total training volume was calculated for all subjects. As training intensity of load was defined as 80% of 1 RM (70% for cable flyes, bicep- and leg- curls), total training volume was calculated as sets × repetitions (reps). Statistical tests revealed no significant difference between groups in LB (Pro: 1858 ± 161 reps, Con: 1818 ± 121 reps; *p* = 0.57) or UB (Pro: 2556 ± 298 reps, Con: 2485 ± 258 reps; *p* = 0.62).

## 4. Discussion

The aim of this study was to investigate the potential effect of insect protein as a dietary supplement to increase muscle hypertrophy and strength gains during prolonged resistance training compared to an isocaloric carbohydrate supplement. The current study shows that protein supplementation from insects after exercise and pre-sleep on training days does not promote greater gains in FBFM and muscle strength after eight weeks of resistance training in young males compared to a non-protein supplemented group consuming on average 1.7 g/kg/day. Previous research indicates that protein supplementation combined with resistance training promotes greater gains in FBFM and muscle strength [26,27]. However, these findings may be questioned due to very small differences in protein intake between controls and protein groups. Moreover, several studies fail to show any significant effect of protein supplementation combined with resistance training [28,29,30,31]. The discrepancy in the literature may be caused by several factors such as the design of the resistance training program, the dietary habits of subjects, supplementation doses, timing and quality, training status, length of study, and sample size.

The overall study design was well tolerated by the subjects, resulting in no dropouts and a high training compliance of 97.6 ± 2.7%. The resistance training program effectively augmented FBFM and 1 RM strength in both groups with no significant differences. A mean hypertrophic response of 2.5 ± 1.5 kg (4.2%) and 2.7 ± 1.5 kg (4.2%) in FBFM for Con and Pro, respectively, is notably high compared to similar studies with protein supplementation [29,32,33]. Since no significant difference between groups existed, the change in FBFM must be attributed to the resistance training program combined with energy supplementation.

Comparable to studies examining strength gains following resistance training for 7–16 weeks [34,35,36,37], the subjects in the present study increased leg and bench press 1 RM muscle strength by 10–24% from pre to post. This is in line with most similar studies on protein supplementation and resistance training for 8–12 weeks, which report strength gains in 1 RM between 12% and 44% [29,30,32,33,38,39,40]; however, a few outliers [26,27,41] appear with strength gains over 100% in 1 RM. The discrepancy between strength gains in the present study and the outliers may be due to subject characteristics and training status. Research indicates that the initial strength increase during resistance training is mainly caused by neural adaptations in the first three to five weeks, after which hypertrophy becomes the dominant factor [42]. Subjects from the present study had an activity level of up to 10 h/week (see “Subjects”) in contrast to less than 2–3 h/week in the studies by Hartman et al. [26] and Josse et al. [41]. This may influence the strength gains caused by neural adaptations and explain how strength gains over 100% in 1 RM can be reported.

The dietary analysis revealed significant difference in protein intake between groups on training days (Con: 1.7 ± 0.3 g/kg/day, Pro: 2.3 ± 0.3 g/kg/day; *p <* 0.05). Recommendations on optimal daily protein intake vary between 0.8 and 1.6 g/kg/day depending on physical activity level, gender, and age [43,44]. Nevertheless, this reflects the fact that subjects in Con consumed an adequate amount of protein during the intervention period. The dietary analysis also revealed a trend towards a difference in total energy intake during the intervention (*p* = 0.10). On non-training days, Con increased total energy intake from 2764 ± 312 kcal (Pre) to 3069 ± 621 kcal (Mid) (*p* = 0.28) and Pro decreased from 3142 ± 747 kcal (Pre) to 2785 ± 441 kcal (Mid) (*p* = 0.2218), which may reflect different changes in FM between groups (Con: 0.2 ± 0.9 kg, Pro: −0.4 ± 1.2 kg; *p* = 0.27) (Figure 2). Moreover, a small decrease in total energy intake in Pro might be of concern, as a study by Friedlander et al. [45] showed that a total positive energy balance is necessary to maintain optimal anabolic processes and attenuate catabolism. Therefore, group differences in energy balance might also explain why no difference in strength and hypertrophy was detected between groups. However, it should be noted that variations within the dietary records may exist because of the relatively short registration periods of three days. Nevertheless, the observed decrease in total energy intake in Pro may also be explained by a higher satiety when consuming the protein bars compared to the carbohydrate bars [46].

The present study had no dietary restrictions before and after training, which may contribute to the fact that no differences in body composition changes were observed between groups. In addition, the time of training was not controlled and we observed that most subjects trained in the late afternoon. This may interfere with optimal timing strategies for protein supplementation, as suggested by Areta et al. [11], as both post-training and pre-sleep supplements were mainly consumed over a short period of time rather than dispersed throughout the day. Furthermore, it must be noted that no monitoring of supplement compliance was performed, which may be a limiting factor of the overall study design. Alternatively, a method of dietary restriction is used in the study by Rindom et al. [47], which required subjects to follow a controlled diet to ensure fixed amounts of macronutrients, in line with the desired study aim. This method is invasive and costly for studies with longer intervention periods. Josse et al. [41] and Hartman et al. [26] used dietary restrictions 2 h before training to ensure differences in macronutrient availability post-training. However, from our perspective these methods do not reflect reality as diet restrictions interfere with subjects’ dietary habits and thereby manipulate the target group. In a report based on data from the Danish national survey of dietary habits and physical activity in 2011–2013 [48], the average protein intake of the adult population accounted for 16% of daily energy intake. This is in accordance with the average habitual protein intake of 15.3% from subjects of the present study, which confirms that the target group is representative of the average Danish adult.

An intervention period of eight weeks with resistance training may seem short compared to similar studies of 10–14 weeks [26,41,49]. According to Tipton and Wolfe [50], it would take up to a year of training to detect the effects of a post-exercise protein supplement on FBFM due to the relatively small effect size compared to resistance training and habitual nutrient intake. A more recent meta-analysis by Cermak et al. [8] reported an effect size of 0.69 kg FBFM after 12 weeks of resistance training with protein supplementation compared with a placebo. Despite only eight weeks of resistance training, we still detected great responses in FBFM and muscle strength; however, no benefits of protein supplementation were observed. In the present study, sample size was determined with the use of data from Kerksick et al. [32], where gains in FBFM were 0.0 kg and 1.8 kg for Con and Pro, respectively. This indicated statistical power, with a sample size consisting of six subjects in each group. However, it seems questionable that a group of young men performing resistance training for 10 weeks with energy supplementation would not gain FBFM when it is commonly known that resistance training alone results in increased FBFM provided that a positive energy and protein balance is maintained [26,27,40,41,51,52]. Therefore, we cannot rule out the possibility that our sample size may have been too small in regard to detecting a difference in muscle growth in response to resistance training between two supplementation groups. However, the present results do not indicate that a larger sample size would elicit any clinically relevant difference in FBFM between groups.

Although we did not detect any benefits of timed protein supplementation during exercise intervention in healthy, well-nourished young men, insect protein may still have value as an alternative protein source. AA profiles by Yi et al. [2] showed that protein from the lesser mealworm (*Alphitobius diaperinus*) equally match the profile of soybean in regards to EAA, although no AA analysis was conducted on the specific protein isolate (Experimental Protein Isolate, Proti-Farm R&D BV, Ermelo, The Netherlands) used in this study. A review by Devries and Phillips [16] examined the difference in quality between whey, casein, and soy protein, and found greater advantages for whey protein due to AA content, digestibility, and bioavailability. While insect protein seems to match soy protein in terms of AA content, digestibility and bioavailability might be more similar to whey as it is also an animal protein. It should be noted, though, that the protein supplementation in the present study was served as a complex baked bar, which may have impaired the digestibility of the protein supplementation. However, blood samples would have to be conducted to support this notion. Moreover, a recent study by Janssen et al. [3] suggested that the Kp factor of 6.25 normally used in the Kjeldahl method might overestimate the total protein content in insects due to the presence of non-protein nitrogen in the insect. An alternative Kp factor of 5.60 ± 0.39 is suggested to be more accurate, which may cause a potential overestimation of the protein content and training dose in the present study (~82%). However, if adjusted this would cause a ~10% overestimation of total protein supplementation, which, for the average subject of ~80 kg, would account for ~6.4g less protein per training day. This would reflect a depreciation of 0.1 g/kg/day for the Pro group (2.3 g/kg/day to 2.2 g/kg/day), which we do not believe would influence the outcome of the investigation. However, this is still considered a limitation, which is why future studies should examine the true protein content before conducting a similar trial. Furthermore, comparative studies are still needed to directly compare the effect of insect protein isolate with other protein sources with regard to promoting muscle growth in active subjects, as this was not possible in the current study due to limited resources.

## 5. Conclusions

With a growing world population and increased need for dietary protein, insect protein should be considered a valuable alternative to other dietary animal protein sources given its environmental profile [53]. Future studies could examine whether insect protein supplementation in groups with a suboptimal protein intake would have a positive effect on skeletal muscle mass and function. For instance, a study on insect protein supplementation in an undernourished elderly population to attenuate loss in FBFM would be of great interest. Furthermore, acute studies are needed to examine the AA content, absorption, and digestibility of insect protein sources in a human trial.

In conclusion, we found that eight weeks of resistance training effectively promote gains in FBFM and muscle strength in healthy young men with a habitual intake of protein corresponding to the general population of young Danish men. However, no difference in morphological adaptations such as hypertrophy or muscle strength was observed between supplementation groups, as hypothesized.

## Figures and Tables

**Table 1:** Resistance training program.

Weeks	Repetitions ^1^	Sets ^2^
1–2	10–12	2
3–4	10–12	3
5–6	8–10	4
7–8	6–8	5

^1^ 90-s rest between sets; ^2^ 3-min rest between exercises.

**Table 2:** Body composition before (Pre) and after (Post; week 9) training.

DXA Results	Con				Pro				Interaction
	Pre	Post	Δ (95% CI)	*p*	Pre	Post	Δ (95% CI)	*p*	*p*
BW (kg) *	80.8 ± 9.1	83.6 ± 9.0	2.8 ^1^ (1.4, 3.2)	<0.01	81.6 ± 8.7	84.0 ± 8.4	2.3 ^1^ (1.4, 3.2)	<0.01	0.54
FM-LB (kg)	8.5 ± 3.1	8.7 ± 3.1	0.2 (0.0, 0.5)	0.10	7.1 ± 1.6	7.0 ± 1.7	−0.1 (−0.4, 0.3)	0.73	0.18
FM-UB (kg) *	8.4 ± 4.3	8.4 ± 4.1	0.0 (−0.3, 0.3)	0.96	6.4 ± 1.9	6.1 ± 2.2	−0.3 (−0.7, 0.2)	0.25	0.34
FM-total (kg) *	17.8 ± 7.4	18.1 ± 7.1	0.2 (−0.1, 0.8)	0.44	14.6 ± 3.5	14.3 ± 3.8	−0.4 (−1.2, 0.5)	0.43	0.27
FBFM-LB (kg)	30.4 ± 2.3	31.4 ± 2.7	1.0 ^1^ (0.4, 1.5)	<0.01	31.8 ± 4.0	33.0 ± 4.0	1.2 ^1^ (0.8, 1.5)	<0.01	0.50
FBFM-UB (kg) *	25.7 ± 2.0	27.3 ± 2.5	1.6 ^1^ (1.1, 2.0)	<0.01	27.6 ± 3.8	29.1 ± 3.7	1.5 ^1^ (0.8, 2.2)	<0.01	0.91
FBFM-total (kg) *	59.7 ± 4.2	62.2 ± 5.2	2.5 ^1^ (1.6, 3.5)	<0.01	63.5 ± 7.9	66.1 ± 7.7	2.7 ^1^ (1.6, 3.8)	<0.01	0.86
BMC (g)	3299 ± 399	3317 ± 383	18 ± (−3, 39)	0.09	3546 ± 378	3555 ± 371	8.5 (−10, 27)	0.37	0.51

BW, body weight; BMC, bone mineral content; Con, control group; FBFM, fat- and bone- free mass; FM, fat mass; LB, lower body; Pro, protein group UB, upper body. Values are presented as mean ± standard deviation (SD), with all delta values as (Δ (95% confidence interval (CI))). Statistical analysis of interaction was by 2 × 3-factor analysis of variance (ANOVA) within groups (Pre and Post) and between groups (control and insect protein); * Data for protein group included only seven subjects due to injuries in upper body; ^1^ Significantly different from pre-intervention (Pre) to post-intervention (Post) within the same group, *p* < 0.01.

**Table 3:** Energy and macronutrient intake before (Pre) and during the intervention (Mid-intervention (Mid)) ^1^.

Nutrition Data	Con (*n* = 6)			Pro (*n* = 9)			Interaction
	Pre	Mid *	*p*	Pre	Mid *	*p*	*p*
Energy intake kcal/day	2764 ± 312	3593 ^2^ ± 621	<0.01	3142 ± 747	3312 ± 441	0.56	0.10
Protein intake g/day	101.7 ± 16.9	128.2 ^2^ ± 16.6	0.01	123.0 ± 24.4	182.5 ^2,3^ ± 15.6	<0.01	0.02
Protein intake g/kg/day	1.3 ± 0.3	1.7 ± 0.3	0.03	1.6 ± 0.3	2.3 ^2,3^ ± 0.3	<0.01	<0.05
Fat intake g/day	104.3 ± 18.2	118.2 ± 39.7	0.44	114.5 ± 33.8	91.6 ± 53.3	0.28	0.18
Fat intake g/kg/day	1.4 ± 0.3	1.55 ± 0.5	0.47	1.4 ± 0.3	1.2 ± 0.7	0.33	0.23
CHO intake g/day	309.8 ± 86.1	443.8 ^2^ ± 56.7	<0.01	366.6 ± 73.9	381.7 ^2,3^ ± 16.5	0.55	0.02
CHO intake g/kg/day	4.0 ± 1.0	5.8 ^2^ ± 0.4	<0.01	4.6 ± 0.9	4.8 ^2,3^ ± 0.2	0.62	<0.01

* Mid-intervention data were calculated as a 3-day mean including one training day’s dietary supplementation. ^1^ All values are mean ± SD. CHO, carbohydrate. Con, control group; Pro, protein group. Statistical analysis was by two-factor ANOVA within groups (Pre and Mid) and between groups (Pro and Con). There was a significant group-by-time interaction (*p <* 0.05) for total daily protein intake, and total daily CHO intake; ^2^ Significantly different from pre- to mid- within the same group, *p <* 0.05; ^3^ Significantly different in group-by-time interaction, *p <* 0.05.

**Table 4:** Subjects’ strength data before (Pre) and after (Post; week 9) training.

Strength Measures	Con *n* = 9 *				Pro *n* = 9 *				Interaction
	Pre	Post	Δ (95% CI)	*p*	Pre	Post	Δ (95% CI)	*p*	*p*
1 RM Leg press (kg) *	175 ± 34	217 ± 41	42 ^1^ (32, 52)	<0.01	181 ± 26	217 ± 22	37 ^1^ (27, 46)	<0.01	0.92
Leg press volume *	1924 ± 491	3140 ± 1175	1216 ^1^ (515, 1915)	<0.01	1957 ± 559	2465 ± 810	508 (−60, 1076)	0.08	0.59
1 RM bench press (kg) *	71 ± 14	85 ± 16	14 ^1^ (10, 17)	<0.01	78 ± 22	86 ± 22	8 ^1^ (5, 12)	<0.01	0.73
Bench press volume *	647 ± 136	648 ± 105	1 (−46, 48)	0.97	645 ± 204	612 ± 173	−33 (−139, 73)	0.54	0.30
MVC Ext. (Nm)	341 ± 56	403 ± 61	62 ^1^ (50, 75)	<0.01	356 ± 71	403 ± 77	47 ^1^ (19, 75)	<0.01	0.84
MVC Flex. (Nm)	157 ± 16	176 ± 13	20 ^1^ (10, 29)	<0.01	173 ± 26	183 ± 29	10 ^1^ (3, 18)	0.01	0.67
CMJ (W)	1027 ± 15	1075 ± 172	48 ^1^ (26, 70)	<0.01	1068 ± 165	1092 ± 139	24 ^1^ (2, 47)	0.03	0.65

1 RM, one repetition maximum; Con, control group; CMJ, counter-movement jump; Ext., knee extension; Flex., knee flexion; Pro, protein group; MVC, maximal voluntary contraction. All values are presented as mean ± SD, with delta values as (Δ (95% CI)) Leg press and bench press volume are calculated as repetitions (reps) × kg. * Data for leg press in Con included seven subjects, and data for bench press in Pro included seven subjects due to injuries during or post-intervention. ^1^ Significantly different from pre- to post- within the same group, *p* < 0.05.

## Abbreviations

1 RM	One repetition maximum
AA	Amino acid
ANOVA	Analysis of variance
BMC	Bone mineral content
BW	Body weight
CMJ	Counter-movement jump
Con	Control group
DXA	Dual-energy X-ray absorptiometry
EAA	Essential amino acid
FBFM	Fat- and bone- free mass
FM	Fat mass
LB	Lower body
MPS	Muscle protein synthesis
MVC	Maximal voluntary contraction
Post	Post-intervention
Pre	Pre-intervention
Pro	Protein group
UB	Upper body
